# Acute stimulation of glycolytic flux in cultured primary astrocytes by the tyrosine kinase inhibitor tyrphostin 23

**DOI:** 10.1186/2193-1801-4-S1-P3

**Published:** 2015-06-12

**Authors:** Eva-Maria Blumrich, Reshma Kadam, Ralf Dringen

**Affiliations:** Centre for Biomolecular Interactions Bremen, Faculty 2 (Biology/Chemistry), University of Bremen, Germany; Centre for Environmental Research and Sustainable Technology, University of Bremen, Germany

**Keywords:** Tyrphostin 23, astrocytes, glycolysis

Tyrphostin 23 (T23) is a well-known inhibitor of protein tyrosine kinases. To investigate potential effects of T23 on the viability and the glucose metabolism of brain cells, we exposed cultured primary rat astrocytes to T23. While the viability and the morphology of the cells were not acutely affected during an incubation of the cultures for up to 4 hours with T23 in concentrations of up to 200 µM, the presence of T23 rapidly stimulated glycolytic flux as demonstrated by a time- and concentration-dependent increase in glucose consumption and lactate release. Maximal effects were observed for incubations with 100 µM T23 which caused a doubling of glucose consumption and lactate production. The stimulation of glycolytic flux by T23 was fully reversible upon removal of the compound. In contrast to T23, the structurally related tyrosin kinase inhibitor tyrphostin 25 did not affect glycolytic flux, nor was the stimulation by T23 substantially affected by the trichloracetate-induced activation of pyruvate dehydrogenase. Further experiments are now required to elucidate the mechanism of T23-induced stimulation of astrocytic glycolysis.

